# The metagenomic landscape of a high‐altitude geothermal spring in Tajikistan reveals a novel *Desulfurococcaceae* member, *Zestomicrobium tamdykulense* gen. nov., sp. nov

**DOI:** 10.1002/mbo3.70004

**Published:** 2024-10-10

**Authors:** Munavvara Dzhuraeva, Khursheda Bobodzhanova, Nils‐Kåre Birkeland

**Affiliations:** ^1^ Center of Biotechnology of the Tajik National University Dushanbe Tajikistan; ^2^ Department of Biological Sciences University of Bergen Bergen Norway

**Keywords:** *Desulfurococcaceae*, geothermal spring, hyperthermophilic archaea, metagenome‐assembled genome, *Zestosphaera tikiterensis*

## Abstract

Metagenomic analysis was conducted to assess the microbial community in the high‐altitude Tamdykul geothermal spring in Tajikistan. This analysis yielded six high‐quality bins from the members of Thermaceae, Aquificaceae, and Halothiobacillaceae, with a 41.2%, 19.7%, and 18.1% share in the total metagenome, respectively. Minor components included *Schleiferia thermophila* (1.6%) and members of the archaeal taxa *Pyrobaculum* (1.2%) and *Desulfurococcaceae* (0.7%). Further analysis of the metagenome‐assembled genome (MAG) from the *Desulfurococcaceae* family (MAG002) revealed novel taxonomy with only 80.95% closest placement average nucleotide identity value to its most closely related member of the *Desulfurococcaceae* family, which is part of the *Thermoproteota* phylum comprising hyperthermophilic members widespread in geothermal environments. MAG002 consisted of 1.3 Mbp, distributed into 48 contigs with 1504 predicted coding sequences, had an average GC content of 41.3%, a completeness and contamination rate of 98.7% and 2.6%, respectively, and branched phylogenetically between the *Ignisphaera* and *Zestosphaera* lineages. Digital DNA‐DNA hybridization values compared with *Ignisphaera aggregans* and *Zestosphaera tikiterensis* were 33.7% and 19.4%, respectively, suggesting that this MAG represented a novel species and genus. Its 16S rRNA gene contained a large 421 bp intron. It encodes a complete gluconeogenesis pathway involving a bifunctional fructose‐1,6‐bisphosphate phosphatase/aldolase; however, the glycolysis pathway is incomplete. The ribulose monophosphate pathway enzymes could be used for pentose synthesis. MAG002 encodes several hydrogen‐evolving hydrogenases, with possible roles as hydrogen sinks during fermentation. We propose the name *Zestomicrobium tamdykulense* gen. nov. sp. nov. for this organism; it is the first thermophilic genome reported from Tajikistan.

## Graphical abstract

Metagenomic analysis of a high‐altitude hot spring in Tajikistan revealed a novel archaeal genus from the family *Desulfurococcaceae*: *Zestomicrobium tamdykulense* gen. nov., sp. nov., as a minor component of the microbial community. The spring was strongly dominated by the genus *Meiothermus*.

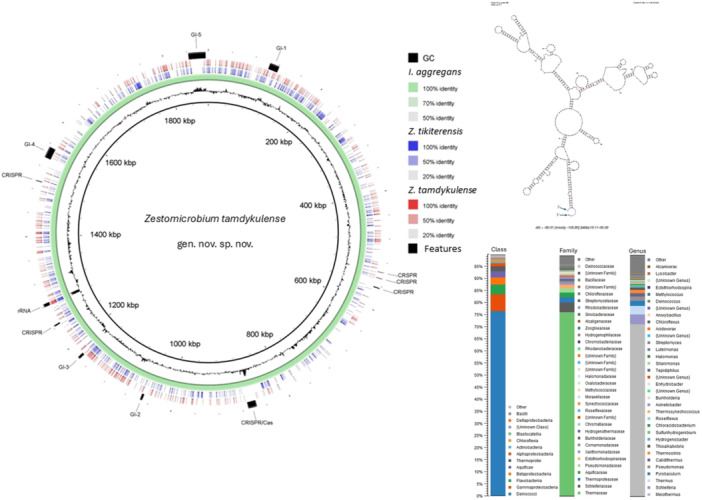

## 1 INTRODUCTION

Extreme environments, such as hot springs, submarine hot vents, salt lakes, soda lakes, and volcanic regions, represent a rich source of extremophilic bacteria and archaea with great potential for use in industrial and biotechnological processes (Egamberdieva et al., 2018; Kuddus, 2021). Thermophilic microbes growing at 50 to 100°C provide many thermostable hydrolytic enzymes such as proteases, lipases, amylases, cellulases, etc. which are exploited in industrial and biotechnological processes globally (Atalah et al., 2019; Kuddus, 2021; Satyanarayana et al., 2013). Terrestrial thermal springs are widely distributed worldwide and vary greatly in temperature, pH, and redox level, which are factors that drive evolution and shape the diversity and community structure of hot spring microbiota (Inskeep, 2013). Thermal springs are classified according to the origin of the water: magmatic water originating in volcanic areas and hot telluric water formed when groundwater passes along deep hot rocks (Arsanova, 2014), which further affects water mineralization and ultimately drives the evolution and diversity of microbial communities (Castelán‐Sánchez et al., 2020).

During the last decade, metagenomics‐based assessments of geothermal environments have boosted our understanding of microbial community structures in these habitats and the mining of novel thermostable enzymes of industrial and biotechnological potential (Chopra et al., 2020; Kuddus, 2021; Kumar, 2023; Nelson, 2020; Strazzulli et al., 2017). Metagenomic analysis of “biological dark matter” has significantly extended the entire tree of life and added a substantial number of novel archaeal and bacterial lineages and phyla with no cultivated representatives (Parks et al., 2017; Tahon et al., 2021). The Genome Taxonomy Database (GTDB; https://gtdb.ecogenomic.org/), based on a comparison of genomes and metagenomes (Parks et al., 2022), currently contains (April 24, 2024; Release 09‐RS2020) 175 bacterial and 19 archaeal phylum‐level lineages, many of which represent uncultivated organisms. Although representatives of the archaeal domain are ubiquitously distributed, with many novel phyla and lineages having been discovered during the last decade, bacterial representatives are still dominant among the cultivated isolates.

We performed the first metagenomics‐based assessment of microbiota in a geothermal spring in Tajikistan. Several high‐quality metagenome‐assembled genomes (MAGs) were reconstructed, including two archaeal MAGs, one of which represented a novel genus belonging to the hyperthermophilic archaeal family *Desulfurococcaceae* and phylum *Thermoproteota*. This family was first described in 1982 as extremely thermophilic, anaerobic, sulfur‐respiring, or fermentative archaea that utilized proteinaceous substrates such as yeast extract or casein (Zillig et al., 1982). *Desulfurococcaceae* currently encompasses 11 validly published genera, with representatives isolated from globally widespread terrestrial hot springs (https://lpsn.dsmz.de/family/desulfurococcaceae), some of which host small obligate parasites belonging to the candidate phylum *Nanoarchaeota* (Huber et al., 2002; St John et al., 2019; Wurch et al., 2016). Thermophilic archaea have not yet been isolated from the Central Asian region, although a metagenomic survey has identified a few archaea in a hot spring in Kazakhstan (Mashzhan et al., 2021).

## 2 MATERIALS AND METHODS

### 2.1 Sampling and sample processing

**Figure A1:**
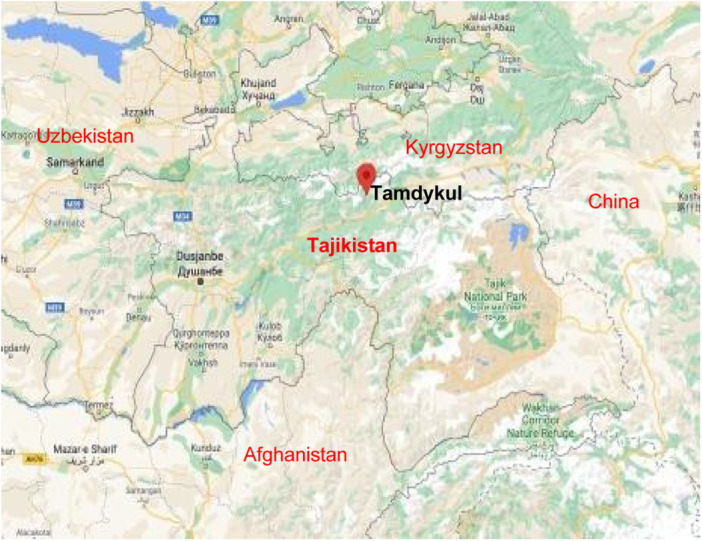
The Location of the Tamdykul geothermal spring.

**Figure A2:**
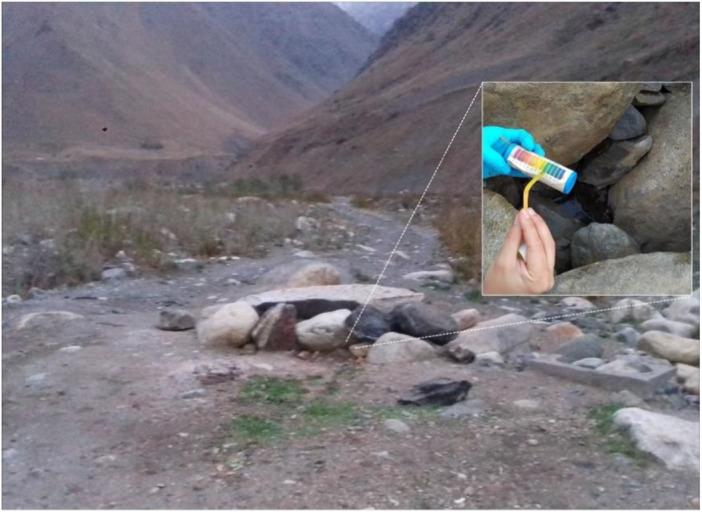
The geothermal spring sampling point located at the bank of the Tamdykul River. The inset shows the exact sampling point.

Water samples were collected from the Tamdykul geothermal field, located 25 km North‐Northwest of the Jirgital district center, in the upper part of the Tamdykul valley near Tamdykul river, at an altitude of 2198 masl (GPS location: 39°25.102′ N, 71°13.103′ E; Figures A1 and A2). The conductivity, temperature, and pH of the outlet water were 1019 μS/cm, 88°C, and 7.4, respectively (Dzhuraeva et al., 2021). The sample was transported to the Tajik National University Biotechnology Center in Dushanbe in 5‐l sterile plastic bottles and processed immediately via first filtering through a 1.2 mm pore‐sized membrane filter to remove debris and particles, followed by filtration through a 0.2 µm pore‐sized membrane filter to entrap the cells. The filter membranes were cut into pieces and stored in RNA later (Sigma‐Aldrich). Membrane pieces were washed by dipping in 0.85% sodium chloride 3–5 times. DNA was then extracted using a DNeasy PowerSoil Kit (Qiagen), as per the manufacturer's instructions. DNA quality was assessed using a Qubit HS dsDNA kit (Life Technologies) and agarose gel electrophoresis. Sampling was first performed in November 2018 (50 l); however, because the DNA yield was not sufficient for shotgun Illumina sequencing, sampling was repeated in September 2019 (75 l). For filtration and DNA extraction, 120 l water was used. DNA from the two samples were pooled and sequenced. The filtered sample was transported to Bergen, Norway, to analyze major and minor elements and anions using an iCAP™ 7600 ICP‐OES Analyzer (Thermo Fisher Scientific) and ion chromatography (IC; Metrohm), respectively, (Table A1) at the ICP‐Laboratory at the University of Bergen (https://www.uib.no/en/geo/111639/icp-laboratory). The major cations found were sodium (179.5 ppm), silicon (50.0 ppm), potassium (7.5 ppm), and calcium (3.8 ppm). The major anions found were sulfate (193.2 ppm) and chloride (35.1 ppm). Nitrates, arsenic, cobalt, chromium, copper, europium, iron, lanthanides, vanadium, yttrium, or zinc were not detected.

### 2.2 Metagenome sequencing and bioinformatics

The metagenome was commercially sequenced at Eurofins Genomics, Constance, Germany (https://eurofinsgenomics.eu/) using HiSeq Illumina technology in the NovaSeq 6000 S2 PE150 XP sequence mode and a NEBNext Ultra II library preparation kit (New England Biolabs). Raw reads were imported to the CLC Genomic Workbench (version 22.0; Qiagen Bioinformatics), paired, and trimmed with Trim Reads followed by taxonomic profiling using the Taxonomic Profiling tool and a custom microbial genome reference database (CLC) downloaded on April 22, 2024. Trimmed reads were assembled and binned using the De Novo Assemble Metagenome and Bin Pangenome by Sequence tools, respectively, as implemented in the Microbial Genomics Module of the CLC Genomics Workbench. Bins were uploaded to KBase (https://www.kbase.us/), quality assessed using CheckM v1.0.18 (Parks et al., 2015), and taxonomically assigned using GTDB‐Tk v2.3.2 (Chaumeil et al., 2022). Annotation was performed using the NCBI Prokaryotic Genome Annotation Pipeline v. 6.1 (https://www.ncbi.nlm.nih.gov/genome/annotation_prok) and the Rapid Annotation Using Subsystems Technology (RAST) server (https://rast.nmpdr.org) (Aziz et al., 2008). Metabolic features were predicted using BlastKOALA (Kanehisa et al., 2016) and RAST. The secondary structure of the 16S rRNA was predicted using Mfold (Zuker, 2003). A 16S rRNA gene sequence‐based phylogenetic tree was constructed using MEGA X (Kumar et al., 2018) and the Maximum Likelihood algorithm. A whole‐proteome‐based phylogenomic analysis was conducted using FastME 2.1.6.1 (Lefort et al., 2015), employing the Type (Strain) Genome Server (Meier‐Kolthoff & Göker, 2019), available at the DSMZ website (https://tygs.dsmz.de). The tree was rooted at the midpoint (Farris, 1972). CRISPR/Cas regions were identified using CISPRCasFinder (Abby et al., 2014; Couvin et al., 2018; Grissa et al., 2007). Genome comparisons using BLAST Ring Image Generator were performed as previously described by Alikhan et al. (2011). Functional annotation to Clusters of Orthologous Groups (COG) was performed using the EggNOG‐mapper (Cantalapiedra et al., 2021; Huerta‐Cepas et al., 2019).

## 3 RESULTS AND DISCUSSION

### 3.1 Metagenomic sequencing, taxonomic profiling, and binning

**Figure 1:**
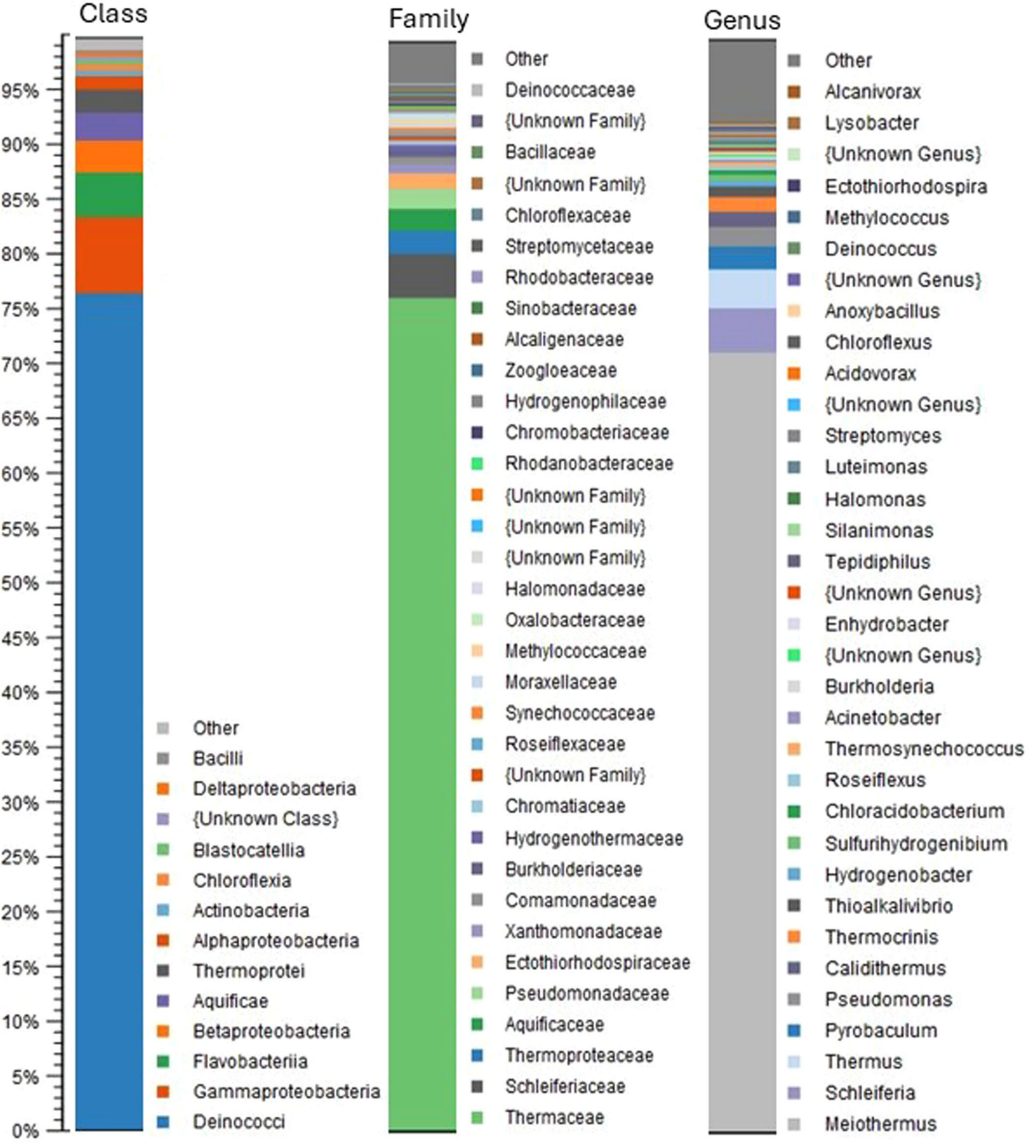
Taxonomic profiling of the Tamdykul hot spring. The relative abundance of prokaryotic taxa at the class, family, and genus levels is shown.

Illumina sequencing of the extracted DNA yielded 9,863,623 reads with a total of 2,959,086,900 sequenced bases. Taxonomic profiling at the Class, Family, and Genus levels using paired reads revealed a high abundance of bacteria belonging to the *Deinococci* class (>75% abundance), most of which were affiliated with the genus *Meiothermus* (~70%), a common heterotrophic inhabitant of hot springs that feeds on a large range of carbohydrates, organic acids, and amino acids (Albuquerque et al., 2009); whereas, members of *Thermus* and *Calidithermus* constituted 4%–5% of the total population (Figure 1). *Pseudomonadota*, including *Gamma*‐, *Beta‐, Alpha*‐, and *Deltaproteobacteria* constituted ~10% of the microbial community with a large diversity of genera, with *Pseudomonas* and chemolithotrophic sulfur oxidizers belonging to genera such as *Thioalkalivibrio* as dominant representatives. The third largest group was *Flavobacteria* (~4%), almost exclusively represented by a member of *Schleiferia*, so far encompassing only one species, *Schleiferia thermophila*, isolated from a hot spring in the Island of São Miguel in the Azores (Albuquerque et al., 2011). Members of the deep‐branching class *Aquificia* and the archaeal class *Thermoprotei* constituted approximately 3% and 2%, respectively. Only members of the metabolically versatile *Pyrobaculum* genus, encompassing both heterotrophic and chemolithoautotrophic hyperthermophilic members with optimal growth temperatures up to 100°C (Huber et al., 1987), were detected among the archaeal group in the taxonomic profiling results. Except for the strong dominance of *Meiothermus*, the microbial community was similar to that of other circumneutral terrestrial hot springs that have been characterized. Minor numbers of possible phototrophic members were identified, including members of *Thermosynechococcus*, *Chromatiaceae*, and *Chloroflexia*. Class *Aquificia* was mostly represented by the chemolithotrophic genera *Thermocrinis* and *Hydrogenobacter*, common inhabitants of hot springs that oxidize hydrogen, thiosulfate, or elemental sulfur.

Following trimming and assembly, a total of 19,712,861 unique base pairs of sequence data were obtained and distributed into 1175 contigs with minimum and maximum lengths of 500 and 505,044 base pairs, respectively. Binning resulted in six high‐quality MAGs with 88.2%–100% completeness and 0%–2.6% contamination (Table 1). These MAGs covered 82.5% of the total metagenomic sequence data and thus represented most of the microorganisms in the sample. MAGs representing the bacterial taxa *Meiothermus cerbereus*, *Aquificaceae*, and *Halothiobacillaceae* constituted the major components, with a 41.2%, 19.7%, and 18.1% share in the metagenome, respectively, including a minor component of *Schleiferia thermophila* (1.6%) (Table 1). Two minor MAGs belonging to archaea were obtained: one representing *Pyrobaculum*, with a 1.2% share of the metagenome, and the other representing *Desulfurococcales* (MAG002), with only a 0.7% share of the metagenome (Table 1). These archaeal groups, which constitute a minor part of the Tamdykul geothermal spring, are common inhabitants of hot spring waters. The closest placement average nucleotide identity (ANI) value for MAG002 was 80.95%, which was the lowest of the six MAGs, substantially below the 95%–96% value that is recommended as a species boundary (Yoon et al., 2017); therefore, this indicates taxonomic novelty within the *Desulfurococcaceae* family (Table 1). MAG002 was the only MAG containing a 16S rRNA gene.

**Table 1:** Characteristics of high‐quality bins from the Tamdykul hot spring.

Bin designation	MAG designation	Length (Mbp)	# contigs	N50	GC content (%)	Completion (%)	Contamination (%)	Closest placement ANI/MSA	16S rRNA gene	Taxonomy	Share in metagenome^a^
Bin001	MAG001	2.53	19	308,548	42.9	100	0	99.6/	0	*Schleiferia thermophila*	1.6
Bin004	MAG004	2.8	109	34,641	61.6	96.6	0	94.1/	0	*Meiothermus cerbereus*	41.2
Bin01	MAG01	1.82	59	73,502	63.4	97.7	0	‐/94.94	0	*Halothiobacillaceae*	18.1
Bin002	MAG002	1.29	48	52,847	41.3	98.7	2.6	80.95/90.34	1	*Desulfurococcales*	0.7
Bin007	MAG007	1.4	136	15,663	43.3	88.2	1.22	98.84/	0	*Aquificaceae*	19.7
Bin003	MAG003	2.1	195	16,469	55.2	99.3	0.74	99.57/	0	*Pyrobaculum* sp.	1.2

^a^ Calculated by mapping raw reads to each individual bin using the Map Reads to Reference tool in CLC.

### 3.2 *Desulfurococcales* MAG002

**Figure 2:**
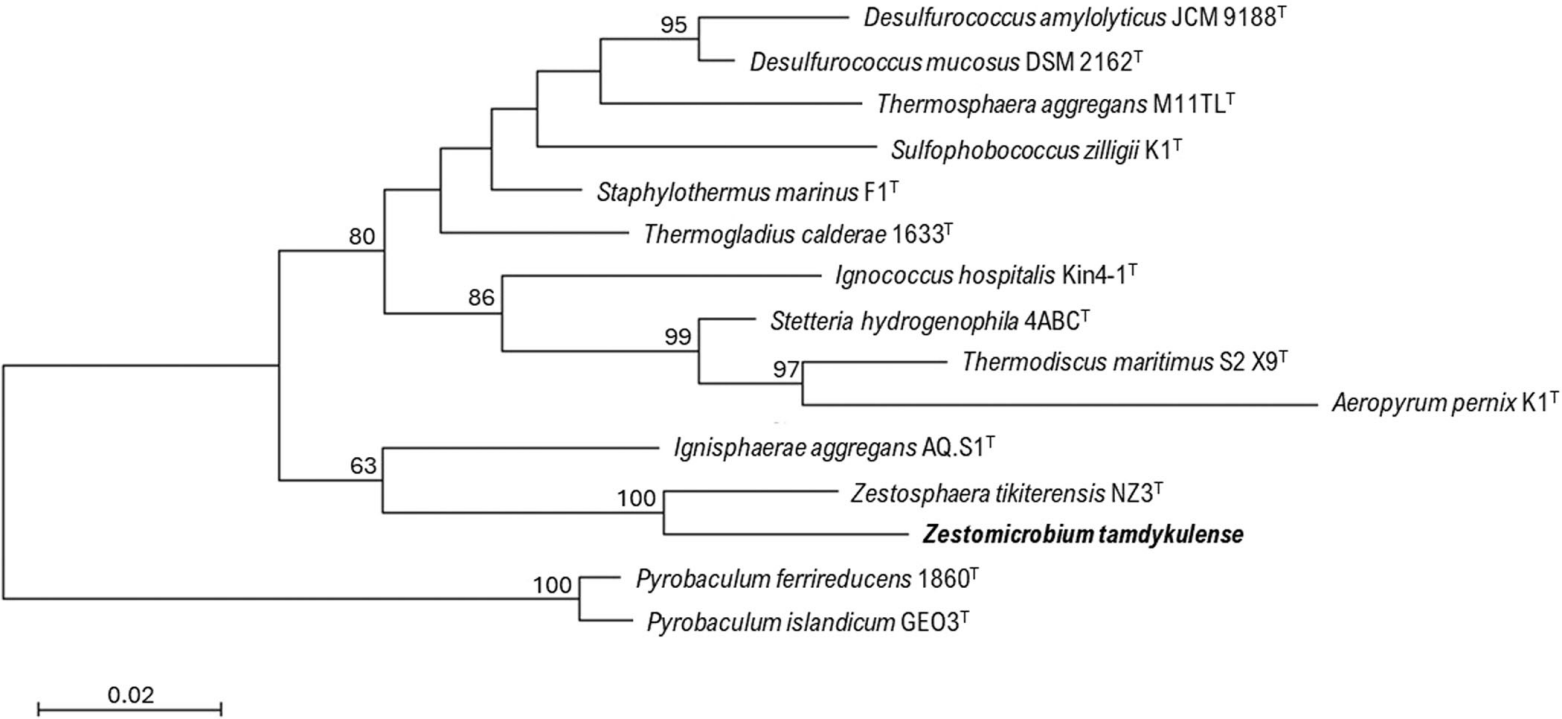
Phylogenetic tree of 16S rRNA gene sequences of the members of family *Desulfurococcaceae* showing the relationship between *Zestomicrobium tamdykulense* (in bold) and other representative type species and strains of this genus. The tree was reconstructed using the Maximum Likelihood method and the Tamura‐Nei model. The tree with the highest log likelihood (‐5122,27) is shown. Bootstrap values (≥63%) are shown as percentages at nodes. All positions containing gaps and missing data were eliminated (complete deletion option). There were a total of 1220 positions in the final data set. Accession numbers: *D. amylolyticus*, AB661712; *D. mucosus*, CP002363; *I. hospitalis*, AJ318042; *I. aggregans*, DQ60321; *S. marinus*, X99560; *S. hydrogenophila*, Y07784; *S. zilligii*, X98064; *T. maritimus*, X99554; *A. pernix*, AB008745; *T. calderae*, CP003531; *T. aggregans*, X99556; *Z. tikiterensis*, MH252993. *P. islandicum* GEO3 (L07511), and *P. ferrireducens* 1860 (CP003098) were used as an outgroup. Bar, 0.02 changes per nucleotide position.

**Figure 3:**
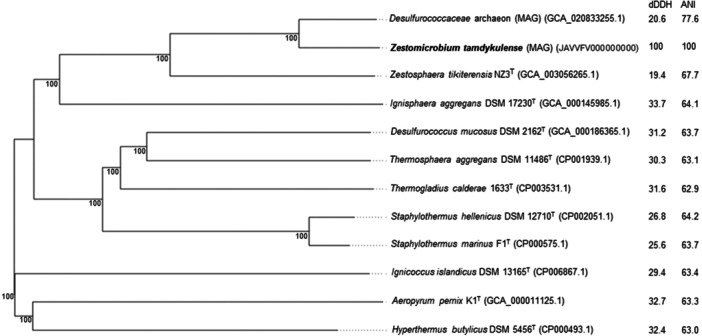
Whole proteome‐based tree of *Zestomicrobium tamdykulense* (MAG2), related type species, and metagenome‐assembled genomes (MAGs) belonging to *Desulfurococcaceae* using the Type (Strain) Genome Server (https://tygs.dsmz.de). The branch lengths are scaled in terms of the Genome Blast Distance Phylogeny (GBDP) distance formula d5. The numbers at branches are GBDP pseudo‐bootstrap support values of 100% from 100 replications, with an average branch support of 100%. The tree was rooted at the midpoint. Pairwise digital DNA‐DNA hybridization (distance formula d4) and average nucleotide identity values are indicated in percentages as compared with those of *Z. tamdykulense*. Genome sequence accession numbers are shown in brackets.

**Figure 4:**
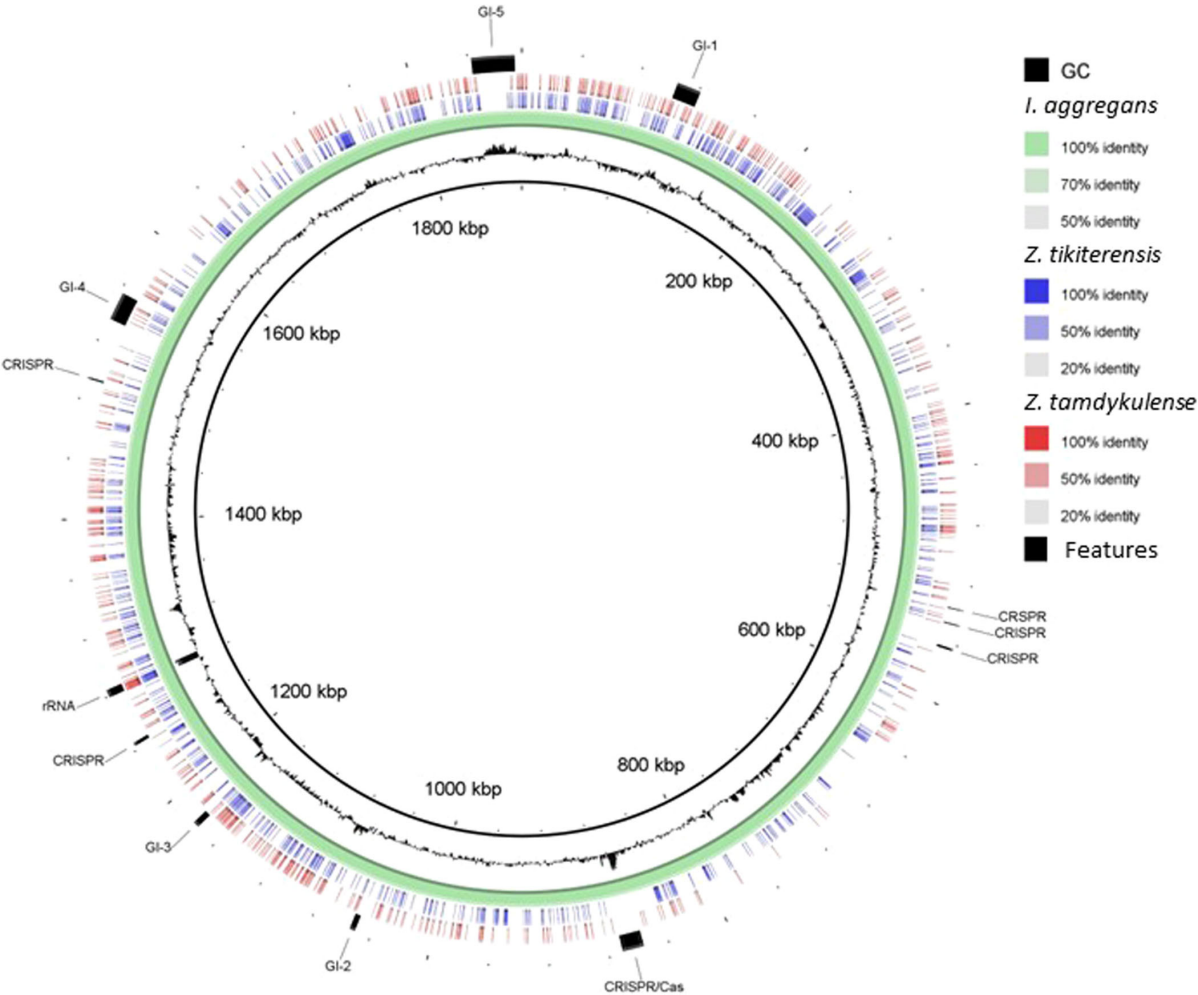
Circular representation of the MAG002 sequence compared to the genome sequences of *Zestosphaera tikiterensis* and *Ignisphaera aggregans*. The complete genome sequence of *Ignisphaera aggregans* was used as a reference. The figure was made using the Blast Ring Image Generator. Rings from inside to outside: G + C content (black); *I. aggregans* AQ.S1^T^ (green); *Z. tikiterensis* NZ3^T^ (blue); MAG002 (red). Genomic islands (GI), rRNA operon, and CRISPR/Cas9 clusters are also indicated in the outermost circle as black arcs. Genome sequence accession numbers are shown in Figure 3.

**Figure A3:**
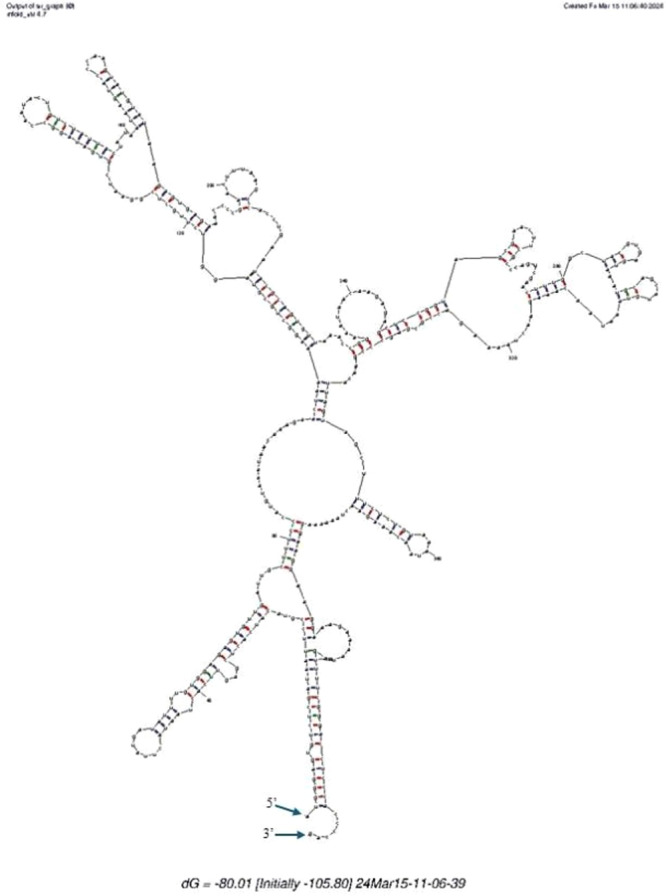
Secondary structure of the 16S rRNA intron of MAG002 as predicted by Mfold. The most energetically favorable conformation is shown. The 5′‐ and 3′‐ends are indicated by arrows.

**Figure A4:**
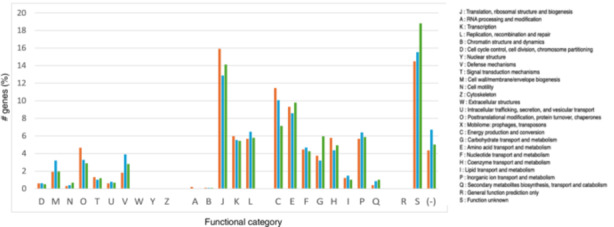
Functional categories of the annotated genes in *Zestomicrobium tamdykulense* (orange), *Zestomicrobium tikiterensis* (blue), and *Ignisphaera aggregans* (green) were classified into clusters of orthologous groups (COG) categories by eggNOG‐mapper v. 2.0. Letter codes are provided on the right.

The 16S rRNA gene sequence of MAG002 most closely matched that of *Ignisphaera aggregans* using BLASTN searches. The gene, containing a small 421‐bp intron, was integrated into a large contig. Introns have previously been found in archaeal rRNA genes, including those of *Desulfurococcales* (Nomura et al., 1998; St John et al., 2019); they sometimes encode homing endonucleases that promote their dissemination through archaeal communities (Aagaard et al., 1995; Jay & Inskeep, 2015). The MAG002 intron did not share any significant sequence similarity with the database entries when analyzed using BLASTN, nor did it encode any homing endonucleases. However, structural prediction revealed potentially extensive secondary structures (Figure A3). After removing the intron from the 16S rRNA gene sequence, the closest BLASTN match was with *Zestosphaera tikiterensis* NZ3 (MH252993.1), sharing 92.52% sequence identity, indicating that MAG002 represented a novel distinct genus, which was clearly below the recommended 16S rRNA gene sequence threshold value of 94.5% for genera (Yarza et al., 2014). The reconstruction of 16S rRNA‐ and proteome‐based phylogenetic trees, including members of all *Desulfurococcaceae* genera, placed MAG002 in a lineage between *Z. tikiterensis* and *I. aggregans* AQ.S1 (Figures 2 and 3, respectively). Genome sequence comparisons showed the highest digital DNA‐DNA hybridization value for *I. aggregans* (33.7%) and the highest ANI value for *Z. tikiterensis* (67.7%), supporting taxonomic novelty (Figure 3). A MAG from a Yellowstone National Park hot spring in the USA formed the closest branch, with an ANI value of 77.6% when compared with MAG002 (Figure 3); therefore, it was sufficiently different to constitute a separate genome species or genus. Genome comparisons using the BLAST ring generator with the *I. aggregans* genome as a reference revealed scattered regions of sequence homology (Figure 4). No CRISPR/Cas regions were found in MAG002, in contrast to the reference strain, AQ.S1, which contained one CRISPR/Cas region and several regions with CRISPR repeats. Genomic islands identified in the reference genome were either absent in MAG002 (e.g., GI–5) or had partial homology (e.g., GI–1 and GI–4). COG analysis revealed a relatively smaller number of genes in the S (function unknown) category in *Zestomicrobium tamdykulense* as compared with *Z. tikiterensis* and *I. aggregans* (Figure A4), which can be explained by a smaller genome of the former organism (1.3 Mbp) in contrast to the 1.8 and 1.9 Mbp genomes of the two latter organisms. This difference in genome size can also explain the larger number of category J (Translation) genes in *Z. tamdykulense* compared with the two other genomes as the reduction of genome size generally increases the fraction of essential housekeeping functions.

### 3.3 Metabolic insights from draft genomes

**Figure A5:**
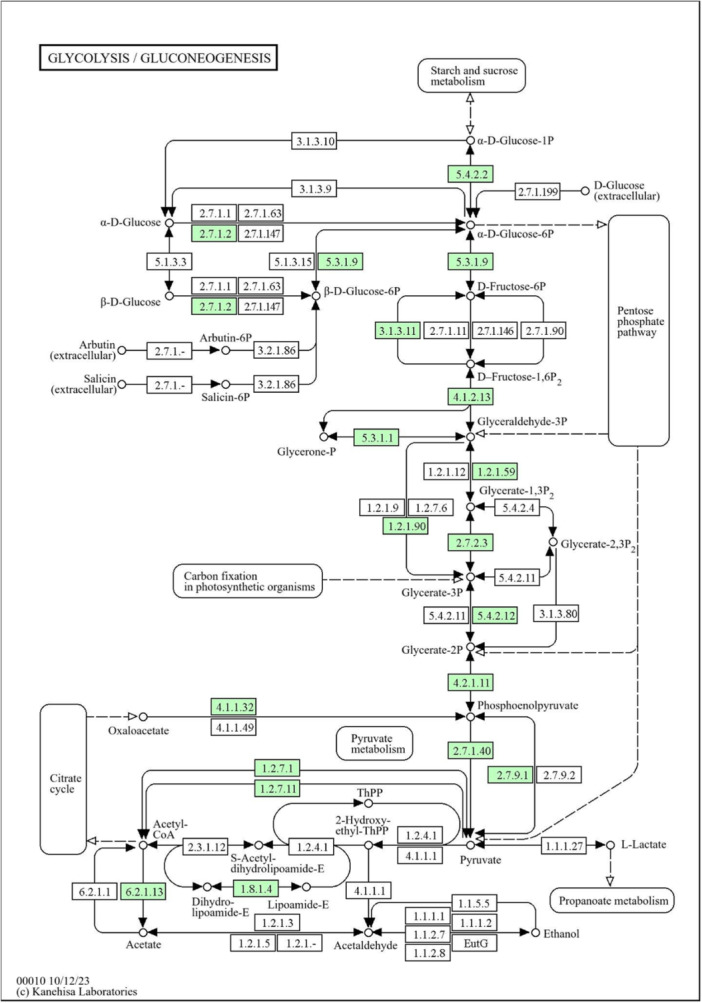
Blastkoala analysis report of the glycolysis/gluconeogenesis pathway in MAG002. Green boxes represent enzymes with enzyme numbers (EC) identified in the metagenome.

To assess the potential metabolic capabilities of *Z. tamdykulense* (MAG002), the draft genome sequence was analyzed using BlastKOALA and RAST. The Embden–Meyerhof–Parnas pathway was found to be incomplete, because genes encoding phosphofructokinases, such as 6‐phosphofructokinase (EC 2.7.1.11), diphosphate‐dependent phosphofructokinase (EC 2.7.1.90), or ADP‐dependent phosphofructokinase (EC 2.7.1.146), which convert d‐fructose‐6‐phosphate (F6P) to d‐fructose‐1,6 P‐bisphosphate (FBP), were absent. However, the gene for the bifunctional fructose‐1,6‐bisphosphate phosphatase/aldolase (EC 3.1.3.11/EC 4.1.2.13) (Aziz et al., 2017; Fushinobu et al., 2011; Say & Fuchs, 2010), which is frequently found in archaea, was identified in MAG002 (Locus tag: QN229_03025). This bisphosphatase dephosphorylates FBP to F6P and also catalyzes the aldolase reaction from FBP to glycerone‐phosphate and glyceraldehyde‐3‐phosphate (Figure A5). The gluconeogenesis pathway is thus complete. However, the bisphosphatase enzyme reaction is irreversible; hence, it renders the glycolysis pathway nonfunctional. *Z. tikiterensis* has been reported to possess incomplete glycolysis and gluconeogenesis pathways, as it lacks genes for FBP phosphatase and FBP aldolase (St John et al., 2019). However, a BLAST search using the MAG002 protein as a query revealed a partial bisphosphatase gene (expectation value: 2*e*‐69) at the end of a contig in the draft genome sequence of strain NZ3, encoding a truncated 258 amino acid protein (locus tag: PUA32121), which probably represents the bifunctional enzyme.

**Figure A6:**
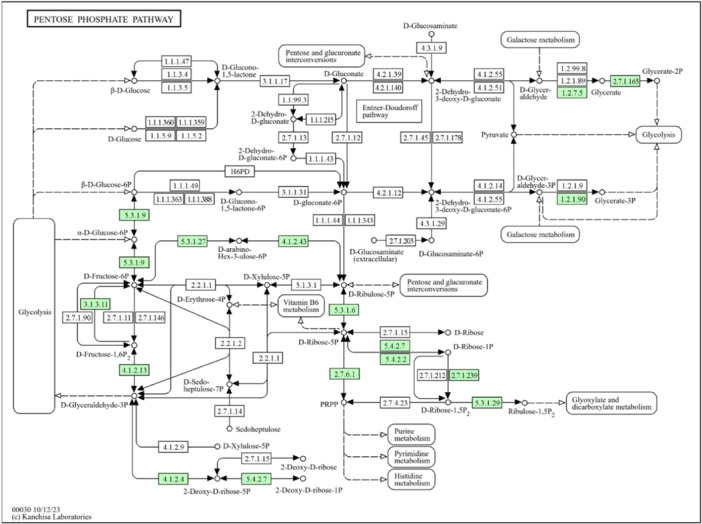
Blastkoala analysis report of the pentose phosphate pathway in MAG002. Green boxes represent enzymes with enzyme numbers (EC) identified in the metagenome.

The pentose phosphate pathway, whose major role is to provide 5‐carbon sugars for nucleoside biosynthesis, is absent in MAG002 and *Z. tikiterensis*. However, both genomes encode the ribulose monophosphate pathway enzymes, 6‐phospho‐3‐hexuloisomerase (EC 5.3.1.27; locus tag: QN229_05875) and 3‐hexulose‐6‐phosphate synthase (EC 4.1.2.43; locus tag: QN229_02210), which makes a shunt from F6P via d‐arabino‐hex‐3‐ulose‐6‐phosphate to d‐ribulose‐5‐phosphate when operating in the reverse direction, which can then be converted further to phosphoribosyl pyrophosphate, a central precursor for purine and pyrimidine biosynthesis (Figure A6). *Z. tamdykulense* assimilates nitrogen from ammonia using glutamate dehydrogenase (EC 1.4.1.2; locus tag: QN229_05895) and the GS/GOGAT system involving sequential action of glutamine synthetase (EC 6.3.1.2; locus tag: QN229_02995) and glutamine oxoglutarate aminotransferase/glutamate synthase (EC 1.4.1.13; locus tag: QN229_01995). The Entner–Doudoroff and the citric acid pathways are absent.

*Z. tikiterensis* encodes two membrane‐bound [NiFe] group 4b hydrogenase clusters, one of which includes a putative catalytic carbon monoxide dehydrogenase subunit (*cooS*), which suggests energy conservation via carboxydotrophic hydrogenogenesis (St John et al., 2019). The *cooS* subunit was not detected in MAG002. Another group 4b hydrogenase cluster includes a Na^+^/H^+^ antiporter, which is also found in a similar cluster in *Z. tamdykulense*, suggesting the use of a Na^+^ gradient for ATP generation using an archaeal‐type ATP synthase in both organisms. The *Z. tikiterensis* genome also encodes a NiFe 3 A hydrogenase cluster group containing a coenzyme F420‐reducing hydrogenase subunit homolog (St John et al., 2019), normally involved in methanogenesis. However, as the F420 biosynthetic pathway is not found in these organisms, this enzyme probably uses another cofactor. A homolog sharing 28% amino acid identity was also detected in *Z. tamdykulense*, but this belonged to a cluster of four genes annotated as sulfhydrogenases (Locus tags: QN229_02275, QN229_02280, QN229_02284, QN229_02290). Sulfhydrogenases are bifunctional 4‐subunit enzyme complexes that evolve hydrogen and reduce elemental sulfur to hydrogen sulfide (EC 1.12.1.5 and EC 1.12.98.4). This enzyme is important for fermentation in the hyperthermophilic archaeon *Pyrococcus furiosus* (Adams et al., 2001).

*Z. tamdykulense* is an amino acid auxotroph as none of the amino acid biosynthesis pathways seem to be complete. This is also the case for purines and pyrimidines except for uracil biosynthesis. Two ABC nucleoside uptake gene clusters and two clusters of oligopeptides/dipeptides ABC transporter systems were identified in the metagenome. A total of 22 genes encoding ATP‐binding proteins for the transport of oligopeptides, dipeptides, and amino acids were found, indicating a high potential for importing a large variety of organic nutrients, adapting to growth in nutrient‐rich environments, and supporting an auxotrophic lifestyle.

## 4 CONCLUSIONS

Metagenomic analysis of the Tamdykul geothermal spring revealed a high abundance of bacteria from the families *Thermaceae*, *Aquificaceae*, and *Halothiobacillaceae*, along with several high‐quality MAGs, including one from a novel genus of the archaeal family *Desulfurococcaceae*. Further analysis of this MAG revealed a 16S rRNA gene sequence containing a 421 bp noncoding intron. The MAG encodes a complete gluconeogenesis pathway involving a bifunctional fructose‐1,6‐bisphosphate phosphatase/aldolase; however, the glycolysis pathway is incomplete. It contains ribulose monophosphate pathway enzymes, which may be used for pentose synthesis. Additionally, it encodes several hydrogen‐evolving hydrogenases with possible roles in fermentation as hydrogen sinks. We propose the name *Z. tamdykulense* gen. nov., sp. nov. for this organism, which is the first reported thermophile genome from Tajikistan. The thermophile genomes presented in this work represent a rich source of novel thermostable enzymes with potential use in agro‐industrial processes and molecular biotechnology.

## AUTHOR CONTRIBUTIONS

**Munavvara Dzhuraeva**: Conceptualization (equal); data curation (lead); formal analysis (equal); investigation (equal); methodology (equal); validation (equal); visualization (equal); writing—original draft (equal); writing—review and editing (equal). **Khursheda Bobodzhanova**: Conceptualization (equal); data curation (supporting); formal analysis (supporting); funding acquisition (equal); investigation (supporting); methodology (supporting); project administration (equal); supervision (equal); validation (supporting) writing—original draft (supporting); writing—review and editing (supporting). **Nils‐Kåre Birkeland**: Conceptualization (equal); data curation (equal); formal analysis (equal); funding acquisition (equal); investigation (equal); methodology (supporting); project administration (lead); supervision (equal); validation (equal); writing—original draft (equal); writing—review and editing (lead).

## CONFLICT OF INTEREST STATEMENT

The authors declare no conflict of interest.

## ETHICS STATEMENT

None required.

## Data Availability

The nucleotide sequence of MAG002 reported and discussed in this paper is deposited at DDBJ/ENA/GenBank under accession JAVVFV000000000: https://www.ncbi.nlm.nih.gov/nuccore/JAVVFV000000000. The version described in this paper is JAVVFV010000000. *Zestomicrobium tamdykulense* SeqCode registry accession: https://registry.seqco.de/registers/r:9-vfcl8f.

## 1

**Table A1:** Chemical composition of the Tamdykul hot spring water.

Elements	(ppm)	Anions	(ppm)
Aluminum	0.035	Bromide	0.92
Boron	1.324	Chloride	35.10
Barium	0.004	Sulfate	193.17
Calcium	3.797		
Lead	0.018		
Lithium	0.595		
Magnesium	0.088		
Manganese	0.008		
Nickel	0.015		
Sodium	197.480		
Strontium	0.119		
Sulfur	83.039		
Silicon	50.028		
Titanium	0.001		
Potassium	7.503		
